# A knock-in/knock-out mouse model of HSPB8-associated distal hereditary motor neuropathy and myopathy reveals toxic gain-of-function of mutant Hspb8

**DOI:** 10.1007/s00401-017-1756-0

**Published:** 2017-08-05

**Authors:** Delphine Bouhy, Manisha Juneja, Istvan Katona, Anne Holmgren, Bob Asselbergh, Vicky De Winter, Tino Hochepied, Steven Goossens, Jody J. Haigh, Claude Libert, Chantal Ceuterick-de Groote, Joy Irobi, Joachim Weis, Vincent Timmerman

**Affiliations:** 1 Peripheral Neuropathy Research Group, Department of Biomedical Sciences and Institute Born Bunge, University of Antwerp, Universiteitsplein 1, 2610 Antwerpen, Belgium; 2 Institute of Neuropathology, RWTH Aachen University Hospital, Aachen, Germany; 3 VIB Center for Molecular Neurology, University of Antwerp, Antwerpen, Belgium; 4 Transgenic Mouse Core Facility, VIB Inflammation Research Center, Gent, Belgium; 5 Department of Biomedical Molecular Biology, Ghent University, Gent, Belgium; 6 Cancer Research Institute Ghent (CRIG), Ghent University, Gent, Belgium; 7 VIB Inflammation Research Center, Ghent University, Gent, Belgium; 8 Laboratory of Neuromuscular Pathology, Institute Born-Bunge and Translational Neurosciences, University of Antwerp, Antwerpen, Belgium; 9 Neurofunctional Genomics, Biomedical Research Institute (BIOMED), Hasselt University/Transnational University Limburg, School of Life Sciences, Diepenbeek, Belgium; 10 Present Address: Mammalian Functional Genetics Laboratory, Division of Blood Cancers, Australian Centre for Blood Diseases, Monash University, Melbourne, VIC 3004 Australia

**Keywords:** Peripheral neuropathy, Myofibrillar myopathy, HSPB8, Autophagy

## Abstract

Mutations in the small heat shock protein B8 gene (*HSPB8/HSP22*) have been associated with distal hereditary motor neuropathy, Charcot–Marie–Tooth disease, and recently distal myopathy. It is so far not clear how mutant HSPB8 induces the neuronal and muscular phenotypes and if a common pathogenesis lies behind these diseases. Growing evidence points towards a role of HSPB8 in chaperone-associated autophagy, which has been shown to be a determinant for the clearance of poly-glutamine aggregates in neurodegenerative diseases but also for the maintenance of skeletal muscle myofibrils. To test this hypothesis and better dissect the pathomechanism of mutant HSPB8, we generated a new transgenic mouse model leading to the expression of the mutant protein (knock-in lines) or the loss-of-function (functional knock-out lines) of the endogenous protein Hspb8. While the homozygous knock-in mice developed motor deficits associated with degeneration of peripheral nerves and severe muscle atrophy corroborating patient data, homozygous knock-out mice had locomotor performances equivalent to those of wild-type animals. The distal skeletal muscles of the post-symptomatic homozygous knock-in displayed Z-disk disorganisation, granulofilamentous material accumulation along with Hspb8, αB-crystallin (HSPB5/CRYAB), and desmin aggregates. The presence of the aggregates correlated with reduced markers of effective autophagy. The sciatic nerve of the homozygous knock-in mice was characterized by low autophagy potential in pre-symptomatic and Hspb8 aggregates in post-symptomatic animals. On the other hand, the sciatic nerve of the homozygous knock-out mice presented a normal morphology and their distal muscle displayed accumulation of abnormal mitochondria but intact myofiber and Z-line organisation. Our data, therefore, suggest that toxic gain-of-function of mutant Hspb8 aggregates is a major contributor to the peripheral neuropathy and the myopathy. In addition, mutant Hspb8 induces impairments in autophagy that may aggravate the phenotype.

**Electronic supplementary material:**

The online version of this article (doi:10.1007/s00401-017-1756-0) contains supplementary material, which is available to authorized users.

## Introduction

Distal hereditary motor neuropathies (dHMN) are a group of clinically and genetically heterogeneous disorders characterized by the degeneration of motor axons in the peripheral nervous system [35]. In dHMNtype II, lower motor neurons are typically involved, whereas sensory functions are mainly spared and symptoms can rapidly evolve into weakness of lower distal muscles and severe neurogenic muscular atrophy [77]. We and others identified two dominant missense mutations targeting the same lysine 141 residue, the K141N (Lys141Asn) and K141E (Lys141Glu) in the highly conserved α-crystallin domain of the small heat shock protein B8 gene (*HSPB8*/*HSP22*) causative of dHMNtype II [24, 43, 76, 77]. Identical and additional mutations at Lys141 were associated with Charcot–Marie–Tooth disease type 2L (CMT2L), which differs from dHMN by its sensory involvement [55, 74], and recently with distal myopathy [25, 34]. In the latter case, patients with K141E or frameshift mutation in HSPB8 presented a progressive myopathy associated with myofibrillar network disruption and rimmed vacuolar pathology reminiscent of myofibrillar myopathy (MFM) hallmarks [17, 47]. Despite the growing interest in the biological role of HSPB8 and the consequence of its mutation, the pathogenesis of the neuromuscular phenotype in patients with mutant HSPB8 remains to be solved.

The HSPB8 is one of the ten members of the small heat shock protein family (sHSP), a group of molecular chaperones known to be up-regulated under heat stress or toxic stress. All the members of the sHSP family share a highly conserved α-crystallin domain and a remarkable ability to form large dynamic oligomers whose composition and stoichiometry regulate their function [36]. The sHSPs participate in the proteome integrity by binding to the hydrophobic regions of misfolded and non-native proteins in stressful conditions. Besides their canonical function in proteostasis, sHSPs have been involved in an increasing number of cellular functions in stress and physiological conditions such as cellular differentiation and proliferation, translation, oxidative stress regulation, cytoskeleton stabilisation, apoptosis, and autophagy [1, 3, 19, 33, 39, 59, 86]. Interestingly, mutations in HSPB1 and αB-crystallin, two other members of the same sHSP family, have also been associated with inherited peripheral neuropathies [11, 24, 27, 48, 63, 68], and distal myopathy, respectively [28, 53, 82]. The reason why mutations in those ubiquitous chaperones affect specifically peripheral nerves and muscle is unclear. A number of papers published by Carra et al. demonstrated how HSPB8 participates in aggregates clearance through its interaction with the Beclin2 Associated Anathogen 3 (BAG3) complex [13–15]. Dimers of HSPB8 bind to the co-chaperone BAG3 which allow the ubiquitination of the to-be-cleared substrate via its interaction with the heat shock protein 70 (HSP70) and CHIP (carboxyl terminus of HSC70-interacting protein) complex. Once ubiquitinated, the substrate is identified by sequestosome-1 (SQSTM1), also known as the ubiquitin-binding protein p62, and engulfed by the autophagosome for degradation in a process called macroautophagy. The involvement of the HSPB8/BAG3 complex in the clearance of aggregates associated with neurodegenerative diseases such as Huntington’s disease, frontotemporal dementia, amyotrophic lateral sclerosis, and spinobulbar muscular atrophy supports a key role of this chaperone-mediated autophagy in the maintenance of neurons in general and motor neurons in particular [14, 16, 22, 65]. Interestingly, the K141N and K141E missense mutations in HSPB8 associated with dHMN and myopathy reduce the ability of HSPB8 to bind to BAG3, and impair the degradation of aggregates via autophagy in vitro and in vivo [12, 50]. The interaction of the HSPB8/BAG3 complex with HSC70 (the cognate form of HSP70) and p62 (p62/SQTM1) is also essential in skeletal muscle maintenance where it promotes the degradation of the actin-crosslinking protein Filamin C damaged by muscle contraction [2, 80]. Accordingly, mutation or deficiency in p62, BAG3, and HSPB8 has been shown to induce distal myopathy associated with rimmed vacuole and myofibrillar disorganisation on muscle biopsy [10, 25, 34, 40, 49, 71]. New roles are also emerging for the HSPB8/BAG3 complex in actin dynamics, spindle orientation, mitosis, and stress granule regulation [30, 31, 81] and for HSPB8 in mitochondria oxidative phosphorylation and membrane potential maintenance [42, 52, 54, 62]. In addition, mutations in HSPB8 can, in vitro, induce HSPB8 aggregation [42], increase its binding to the RNA helicase Ddx20 [73], and several heat shock proteins [29, 42], as well as impair the ability of primary neurons to form neurites in vitro [41]. It is, therefore, difficult to pinpoint to a main function altered by mutant HSPB8 which could be responsible for the neuropathic and myopathic phenotypes. In this study, we developed a new transgenic mouse model using the endogenous Hspb8 promoter that allowed the generation of mutant Hspb8 knock-in (KI) lines as well as Hspb8 functional knock-out (KO) lines to achieve better understanding of HSPB8-related neuromuscular pathogenesis.

## Materials and methods

### Ethics statement

All mouse experiments were carried out with approval of the Ethical Committee for Laboratory Animals (University of Antwerp). Mice were housed under the care of the Animal Facility Interfaculty Unit, which is accredited by the Association for Assessment and Accreditation of Laboratory Animals. All experiments were performed on adult female Hspb8^K141N/K141N^ (knock-in, KI); Hspb8^−/−^ (knock-out, KO); and Hspb8^+/+^ (wild type, WT) mice; except when otherwise stated.

### Generation of the HspB8 targeting vector

A single targeting vector was generated to produce Hspb8^K141N^ KI and Hspb8 KO transgenic mouse lines. Briefly, the BAC clone bMQ-191J17 (Geneservice, Cambridge, UK) containing the full length mouse *Hspb8* genomic DNA was used as starting material. The Neomycin (*Neo)* selection gene, floxed by Flp recombinase target (*FRT*) sites and followed by a *loxP* site, was retrieved from a pPGK-*loxP*FRT-*Neo*-FRT plasmid (gift from D. J. van Hengel, VIB Department for Molecular Biomedical Research, Ghent). Through PCR, a *loxP* site upstream of exon 2 was added, as well as specific restriction sites to allow further cloning, Southern blot analysis, and genotyping. The restriction enzymes used were *Aat*II, *Bam*HI, *Eco*RV, *Hin*dIII, *Mlu*I, *Not*I, *Sac*II, *Sex*AI, and *Xho*I (New England Biolabs, Ipswich, MA, USA). In a first step, all fragments were cloned individually in a pCR2.1_TOPO vector (Life Sciences, Little Chalfont, UK). Next, in vitro mutagenesis was performed to insert the K141N (c.423 G > C) mutation in the exon 2 of the *Hspb8* gene. The final targeting vector contained (1) the K141N point mutation in exon 2 of the *Hspb8* gene; (2) the *Neo* gene cassette, downstream of *hspb8 exon 2*; (3) two *loxP* sites to allow the excision of exon 2 and the *Neo* cassette to generate a functional Hspb8 KO mouse line.

### Chimeric mouse generation and breeding

The linearized targeting vector was electroporated in G4 embryonic stem cells (G4 ES, Mount Sinai, Toronto, CA) [32]. Clones were selected for resistance to neomycin and screened for correct targeting by PCR, Multiplex Amplicon Quantification (Multiplicom, Antwerp, Belgium) and Southern blot. Correctly targeted cells (from 29/A10 ES cell clone) were aggregated with outbred Swiss morula, which were then implanted into pseudopregnant Swiss mice. The ES cell manipulations and transgenic mouse generation were performed by T.H., S.G., and J.J.H. This led to the generation of a transgenic line having the K141N mutation integrated in the exon 2 of the endogenous mouse *Hspb8* gene. Transgenic mice from the F2-generation were backcrossed on a C57BL/6J genetic background. Homozygous Hspb8^K141N/K141N^, heterozygous Hspb8^K141N/+^ transgenic mice, and wild-type littermates were obtained by crossing heterozygous Hspb8^K141N/+^ lines. Heterozygous Hspb8^K141N/+^ lines were also crossed with Sox2-Cre lines to generate functional Hspb8 KO mice after exon 2 excision by Cre-lox recombination and obtain heterozygous Hspb8^−/+^, homozygous Hspb8^−/−^, and wild-type littermates.

### PCR analyses and sequencing

ES screening and genotyping of transgenic animals was performed by PCR on a Veriti 96 well Thermal Cycler (Applied Biosystems, Thermo Fisher Scientific Inc., Waltham, MA, USA) using 4 primer sets. The primers for the distinction of the WT and KI allele were as follows: LoxP_Fw, ATCTTGAAGCATTGAAGCAAGG (forward primer) and LoxP_Rv, TATTGAAATACGGACTGAGTGGG (reverse primer). The PCR reaction resulted in a 188 bp fragment corresponding to the WT allele and/or a 235 bp fragment corresponding to the KI allele. The KO allele was detected by an amplicon of 390 bp using the following primers: KO_Fw CAGCATCTTGAAGCATTGAAGC and KO_Rv AGCACAAGGGTCCATATACTCCAG. The primers for the detection of the CRE recombinase transgene were CRE5 ATGTCCAATTTACTGACCG and CRE3 CGCCGCATAACCAGTGAA. The genotype was validated by sequencing exon 2 of the *Hspb8* mouse gene after amplification by PCR using the following primers: Hspb8_exon2_Fw GGAAGTTAGGGAGCAGGTGTCC and Hspb8_exon2_Rv GGAAGTTAGGGAGCAGGTGTCC. The PCR contained standard 10× PCR buffer, 50 mM MgCl_2_ (for the KO and Cre PCR) or 1 M betain (for Exon2 and LoxP PCR), 10 mM dNTPs, 0.10 µM of each primer, and 1 unit of Platinum *Taq* Polymerase (Clontech Laboratories, Mountain View, CA, USA) for the KO and Cre PCR. We used Titanium *Taq* polymerase (Clontech Laboratories, Mountain View, CA, USA) to perform the Exon 2 and LoxP PCR on 100 ng genomic DNA isolated from mouse ear or tail biopsies. The PCR conditions for amplification of Exon 2, LoxP, and KO were as follows: initial denaturation for 5 min at 95 °C followed by 35 cycles of 45 s denaturation at 95 °C, 45 s primer annealing at 68 °C (for the Exon2 PCR), 66 °C (for the LoxP PCR), or 60 °C (for the KO PCR) and a final extension of 5 min at 68 °C (for the Exon2 and Lox PCR) or 72 °C (for the KO PCR). The Cre PCR condition was as follows: initial denaturation for 2 min at 95 °C followed by 40 cycles of 30 s at 95 °C, 1 min at 58 °C, and 1 min at 72 °C, and a final extension of 10 min at 72 °C. PCR products were detected on 2 or 3% (for the LoxP PCR product) agarose gels. The genotype was validated by sequencing the exon 2 of the mouse *Hspb8* gene. Sequencing was performed on purified DNA using the BigDye^®^ Terminator v3.1 Cycle Sequencing Kit (Applied Biosystems, Thermo Fisher Scientific Inc., Waltham, MA, USA) and separated on an ABI3730xl DNA Analyser (Applied Biosystems, Thermo Fisher Scientific Inc., Waltham, MA, USA). Resulting DNA sequences were aligned and analysed with the CLC Main workbench software.

### Behavioural phenotype of the mice

All behavioural experiments were carried out according to the recommendation of the Ethical Committee for Laboratory Animals (University of Antwerp). The motor and sensory functions were assessed at 3, 6, 9, 12, and 18 months of age in adult female mice. The behavioural assessment was performed by an experimenter blind to the mice genotype.

#### Tail suspension test

Each mouse was lifted up by the tail at a height of approximately 20 cm and the hind limb spreading reflex versus clasping behaviour was assessed.

#### Accelerating Rotarod

The locomotor performance was assessed with a five station Rotarod Treadmill for mouse (ENV-575 M, Med associates Inc., St Albans, VT, USA) according to the manufacturer’s instructions. Briefly, the mice were put in separated sections on an accelerating rotating rod (4–40 rpm over a 300-speriod) and the latency to fall was recorded. The time spent clinging on the rotating rod without walking was subtracted from the final score. Each mouse was trained for 5 consecutive days before each test.

#### Grip strength test

Four-limb grip strength was measured using the Bioseb grip strength tester BIO-G3S (Bioseb, Vitrolles, France). Mice were placed on a grid accessory and pulled firmly backwards by the tail, provoking a grip response. The maximum force exerted on the grid was recorded on the apparatus. The final score was determined by averaging the strength of three trials.

#### Footprint analysis

The analysis of the mice hind paw footprints was performed as previously described [85]. Briefly, the hind feet of the mice were coated with non-toxic black ink, so that the mice left a trail of footprints as they walked along a 50-cm-long, 10-cm-wide runway. The toe spreading (distance between the first and last toe) and the plantar length (distance between the tip toe and the heel) of three consecutive steps were measured and averaged.

#### Hot plate test

The sensitivity to heat was measured using the IITC Life Science Hot plate analgesia meter (IITC Inc. Woodlands Hills, CA, USA) according to the manufacturer instructions. Briefly, each mouse was dropped in a bottom-less glass container placed on a platform heated at 52 °C. The temperature was chosen according to the mouse strain to produce a slight discomfort without inducing pain or injury. The latency before showing a sign of discomfort in the hind paws (licking, fast removal or jump) was recorded. Mice that did not show any sign of discomfort after 20 s were removed to prevent injury. The final score was determined by averaging the reaction times of three trials.

### Nerve conduction studies

Nerve conduction studies were performed using the Neuro-EMG-Micro system (Neurosoft, Ivanovo, Russia). Subdermal 0.4-mm electrodes were used for stimulation and recording on anesthetized mice (5% isoflurane). Compound muscle action potential amplitudes (CMAPs) were measured by placing the stimulating electrodes at the sciatic notch and the recording electrodes above the gastrocnemius muscle. The CMAPs were measured at supramaximal stimulation. Three consecutive recordings were performed. The highest recorded amplitude and the associated latency were used as final scores.

### Tissue harvesting and processing

After inhalation of a lethal dose of CO_2_, mice were dissected and the sciatic nerve, spinal cord, and gastrocnemius muscles were harvested, snap frozen in liquid nitrogen, and stored at −80 °C for subsequent protein or mRNA analysis. For microscopy analysis, the tibial nerve (distal to the sciatic nerve trifurcation) and gastrocnemius were dissected and fixed in a 3.9% glutaraldehyde for 48 h at room temperature. For enzymatic staining, freshly dissected gastrocnemius was snap frozen in cold methyl butane, sliced in 9 µm cross-sections, and stored at −80 °C until further processing. For staining on fixed sections, mice received a lethal dose of xylazine and ketamine mixture before being transcardially perfused with 4% paraformaldehyde (PFA). The spinal cord, sciatic nerve, and gastrocnemius muscle were harvested, post-fixed in 4% PFA overnight, and stored in 30% sucrose phosphate-buffered saline (PBS) solution.

### Histology

#### Morphometric study on epoxy semi-thin sections and EM analysis

Nerve and muscle samples were stained with unbuffered aqueous 1% osmium tetroxide, dehydrated, and embedded in araldite epoxy resin. Semi-thin sections were cut on an ultra-microtome, stained with 1% toluidine blue, and examined by light microscopy. Images of toluidine blue sections were captured on an Axioskop light microscope (Zeiss, Oberkochen, Germany) equipped with a CCD UC30 camera (Olympus Inc., Tokyo, Japan). Morphometrical evaluation of the axon number in the tibial nerve was performed in sets of images covering the total nerve cross-sectional area using the software ImageJ [70]. The large and medium myelinated axons were manually counted and reported to the nerve area. In addition, samples were analysed using a semi-automatic method to obtain the myelinated fiber size distribution. To this end, images with overlapping regions of the entire nerve were acquired at 40× magnification (plan achromat objective, pixel size = 0.137 µm) and stitched together using the grid/collection stitching plugin [60] in Fiji software [69]. All stitched images were batch-processed with an ImageJ macro to measure the diameter of all individual axons with an automatic segmentation procedure using the Fiji plugin find connected regions after contrast normalization and unsharp mask, and followed by particle size filtering with the particles analysis tool. Transmission electron microscopy (TEM) of the glutaraldehyde-fixed, resin embedded tissue was performed as previously described [9]. All semi-thin and EM analyses were done with biological triplicates (*n* = 3).

#### Staining and immunohistochemistry (IHC)

The fixed spinal cord lumbar enlargement (containing the neurons innervating the lower limbs) was sliced in 14 µm cross sections and stained with cresyl violet to allow neuron quantification. The number of large neurons in the ventral horn of the spinal cord was manually counted using the software image J [70]. Gastrocnemius sections were processed for hematoxylin/eosin and ATPase stainings or immunostaining using the following antibodies: Hspb8 (ab79784, Abcam, Cambridge, UK), αB-crystallin (ADI-SPA-222-D, Enzo life science, Farmingdale, NY, USA), and desmin (M076029, Dako, Agilent, Santa Clara, CA, USA). The fixed sciatic nerve was sliced in 7 µm longitudinal sections and immunostained for Hspb8 and BAG3 (A302-806A-M, Bethyl Laboratories, Montgomery, TX, USA). Immunohistochemistry was performed as previously described [8]. Briefly, after blocking endogenous peroxidases and unspecific epitopes, sections were incubated overnight at 4 °C with the primary antibody, 1 h at room temperature with a horse-radish peroxidase (HRP)-conjugated secondary antibody (Jackson Immuno Research Laboratories Inc., West Grove, PA, USA) and developed using 3,3′-diaminobenzidine (DAB). Sections were counterstained with hematoxylin. Images were taken on an Axioskop light microscope (Zeiss, Oberkochen, Germany) equipped with a CCD UC30 camera (Olympus Inc., Tokyo, Japan). All stainings were done on biological triplicates (*n* = 3).

### Western blot analysis

Samples were homogenized in RIPA buffer (50 mM Tris, 150 mM NaCl, 1% NP40, 0.5% Sodium deoxycholate, and 0.1% Sodium Dodecyl Sulphate) complemented with protease inhibitors (Complete, Roche Diagnostics, Basel, Switzerland) and phosphatase inhibitors (PhosStop, Roche, Basel, Switzerland). Equal concentrations of proteins, as determined by the BCA protein assay (Thermo Fisher Scientific, Waltham, MA, USA), were mixed with NuPAGE LDS sample buffer, heated to 95 °C for 10 min, and then separated by NuPAGE Bis–Tris gel (4–12% polyacrylamide), electro-transferred (XCell SureLock, Invitrogen, Thermo Fisher Scientific, Waltham, MA, USA) onto Nitrocellulose membrane and immunoblotted. Briefly, membranes were blocked in 5% non-fat dry milk in PBS-Tween (0.1%) for 1 h at room temperature and incubated overnight at 4 °C with a primary antibody. The membranes were incubated with HRP-linked secondary antibody treated with Enhanced Chemiluminescence ECL Plus kit reagents (Thermo Fisher Scientific, Waltham, MA, USA) and imaged with ImageQuant imager (GE Healthcare, Wauwatosa, WI, USA). The following antibodies were used: Hsp22 (3059, Cell Signaling, Danvers, MA, USA), N-terminal Hsp22 (SAB2101100, Sigma-Aldrich, St Louis, MO, USA), BAG3 (A302-806A-M, Bethyl Laboratories, Montgomery, TX, USA), LC3B (L7543, Sigma-Aldrich, St Louis, MO, USA), p62 (sc-25575, Santa Cruz Biotechnology Inc., Dallas, TX, USA), GAPDH (GTX627408, Gene Tex Inc., Irvine, CA, USA), and the following HRP-conjugated secondary antibodies: anti-mouse IgG1 (1070-05, Southern Biotec, Birmingham, AL, USA), anti-mouse IgG2b (1090-05, Southern Biotec, Birmingham, AL, USA), and anti-rabbit (W401B, Promega, Madison, WI, USA). Western blots were quantified using ImageJ [70] software as previously described [39]. Briefly, band intensity was determined by the ‘mean pixel gray’ values after Gaussian blur and background subtraction. The ‘mean pixel gray’ value of each protein of interest was then normalized to the ‘mean pixel gray’ value of the loading control and presented as relative quantities. All western blot analyses were run with biological triplicates (*n* = 3).

### Quantitative RT-PCR

The frozen tissue was homogenized in Trizol and total RNA was extracted using the RNeasy lipid tissue kit (Qiagen, Venlo, The Netherlands) according to the manufacturer’s protocol. Possible residual DNA contamination was eliminated by DNase treatment (Ambion, Thermo Fisher Scientific Inc., Waltham, MA, USA). RNA was then converted into cDNA using the SuperScript^®^ III reverse transcriptase (life technologies, Carlsbad, CA, USA). Real-time quantitative polymerase chain (RT-qPCR) reactions were done with 10 ng cDNA diluted in SYBR Green mix (Life Technologies, Carlsbad, CA, USA) and run on a ViiA 7 Real-Time PCR System (Applied Biosystems, Thermo Fisher Scientific Inc., Waltham, MA, USA) with gene-specific primers (mHspb8 5′-AGACCCCTTTCGGGACTCA-3′/5′-GGCTGTCAAGTCGTCTGGAA-3′). Primers were designed making use of Primerbank (http://www.pga.mgh.harvard.edu/primerbank). One sample from each target was used as no-RT control to attest the absence of genomic DNA contamination. Relative gene expression was calculated using the ΔΔC_T_ method [37] and normalized with geometric mean of at least three housekeeping genes. All quantitative RT-PCR analyses were run with biological triplicate.

### Statistical analysis

Statistical analyses were performed using GraphPad Prism version 6 (GraphPad, La Jolla, CA, USA). Differences between genotypes over different ages were tested using two-way repeated-measures ANOVA and Tukey’s post hoc analysis. Comparisons of two data sets were analysed by Student’s *t* test. Values are expressed as mean ± SD. Statistical significance was set at *p* < 0.05. All analyses were performed blind to the genotype of the mice.

## Results

### Generation of the Hspb8 KI and KO lines

**Fig. 1:**
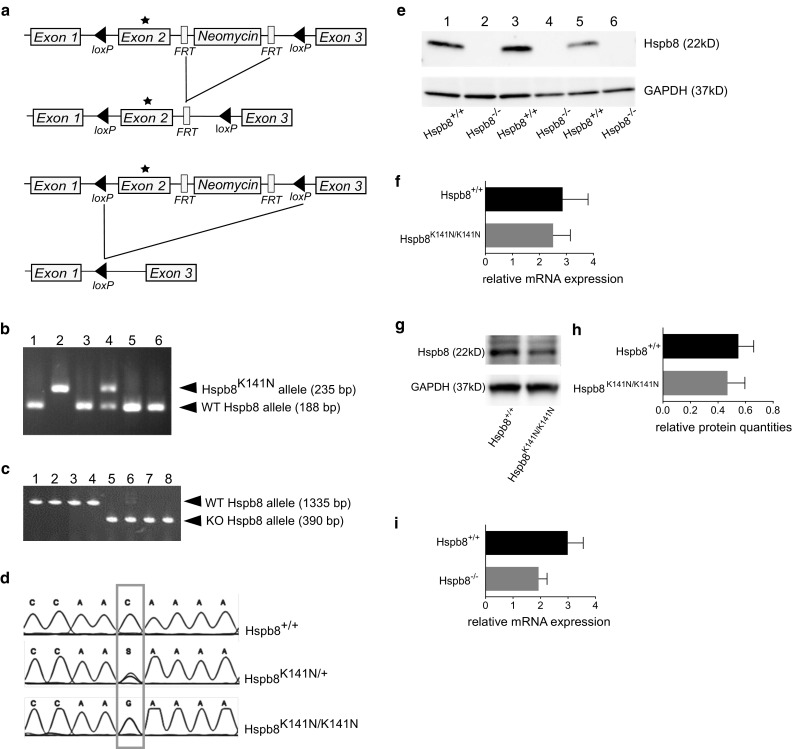
Generation and validation of the Hspb8^K141N^ KI and Hspb8 KO lines. **a** Schematic representation of the mouse Hspb8 allele showing the Neomycin gene (*Neo*) flanked by two *FRT* sites upstream of exon 2. The exon 2 and the *Neo* gene are floxed by two *loxP* sites. Expression of the FLP recombinase allows excision of the *Neo* gene cassette, while expression of Cre recombinase allows Cre-*loxP* recombination and excision of the exon 2 of the *Hspb8* gene as well as the *Neo* cassette. **b** Photograph showing the products of the Exon 2_loxP genotyping by PCR. The 188 bp and 235 bp amplicons resulted from the amplification of the wild-type (WT) and mutant Hspb8 alleles, respectively. *Lanes 1, 3, 5*, and *6* represent genotypes of WT littermates, *lane 2* of a homozygous KI animal, and *lane 4* of a heterozygous KI animal. **c** Photograph showing the products of the KO genotyping by PCR. The 1335 and 390 bp amplicons resulted from the amplification of the wild-type *Hspb8* allele and the allele lacking exon 2, respectively. The *lanes 1–4* represent genotypes of WT animals; *lanes 5–8* of homozygous KO animals. **d** Sequencing of the exon 2 of the mouse *Hspb8* gene showing the presence of the point mutation in a single allele of an heterozygous and homozygous animal. **e** Representative western blot analysis of mouse Hspb8 protein level in the sciatic nerve of 2-month-old KO Hspb8^−/−^ mice (*lanes 2*, *4,* and *6*) and WT littermates (*lanes 1*, *3* and *5*) showing that only the protein lysate from WT produces a band. GAPDH is used as a loading control. **f** Relative quantities (ΔΔC_T_) of Hspb8 mRNA in the spinal cord of 2-month-old Hspb8^K141N/K141N^ and Hspb8^+/+^ mice. Values are expressed as mean ± SD, *N* = 3. **g** Representative western blot analysis of Hspb8 in the spinal cord of 2-month-old Hspb8^K141N/K141N^ and Hspb8^+/+^ mice and quantification of Hspb8 bands normalized on GAPDH (**h**). Values are expressed as mean ± SD, *N* = 3. **i** Relative quantities (ΔΔC_T_) of Hspb8 mRNA in the spinal cord of 2-month-old Hspb8^−/−^ and Hspb8^+/+^ mice. Values are expressed as mean ± SD, *N* = 3

The transgenic Hspb8 KI and KO mouse lines were generated using a single targeting vector encompassing K141N (c.423G > C) point mutation, responsible for dHMN [43], integrated in exon 2 of the endogenous mouse *Hspb8* gene. We developed a gene targeting construct containing the mutated exon 2, a neomycin resistance (*Neo*) gene cassette downstream of exon 2 flanked by two *FRT* sites and two *loxP* sites (one situated upstream of exon 2 and the other downstream of *Neo*) (Fig. 1a). Male chimeras were used for germline transmission and F1-generation mice were backcrossed to the C57BL/6J line to generate a stable colony carrying the Hspb8^K141N^ allele. Hspb8^K141N/+^ mice were bred to generate Hspb8^K141N/K141N^, Hspb8^K141N/+^, and WT littermates (Hspb8^+/+^). The functional KO mouse lines were activated by excision of exon 2 following Cre-lox recombination by crossing Hspb8^K141N^ mice with Sox2-Cre mice [51] (Fig. 1a). The mice genotype was identified by PCR using specific primers to detect Cre recombinase and to differentiate the WT allele (188 bp) from the KI (235 bp) allele (Fig. 1b), and to differentiate the WT or KI alleles (1335/1377 bp) from the KO allele (390 bp) (Fig. 1c). The presence of the K141N point mutation was further validated by Sanger sequencing (Fig. 1d). The homozygous KI mice had normal Hspb8 mRNA and protein levels in the spinal cord, where the protein is highly expressed (Fig. 1f–h), suggesting that the point mutation is not affecting the transcription or the protein stability in this tissue. In the functional KO mice, the excision of Hspb8 exon 2 did not significantly affect its mRNA expression (Fig. 1i). However, the protein could not be detected by western blot using an antibody targeting an epitope coded by the exon 1 of the *Hspb8* mouse gene (Fig. 1e). The generated transgenic mouse lines had a normal lifespan and weight. Their feeding, grooming, and mating behaviours were comparable to those of C57bl/6 wild-type mice.

### Hspb8^K141N/K141N^ mice develop a progressive defect in motor functions

**Fig. 2:**
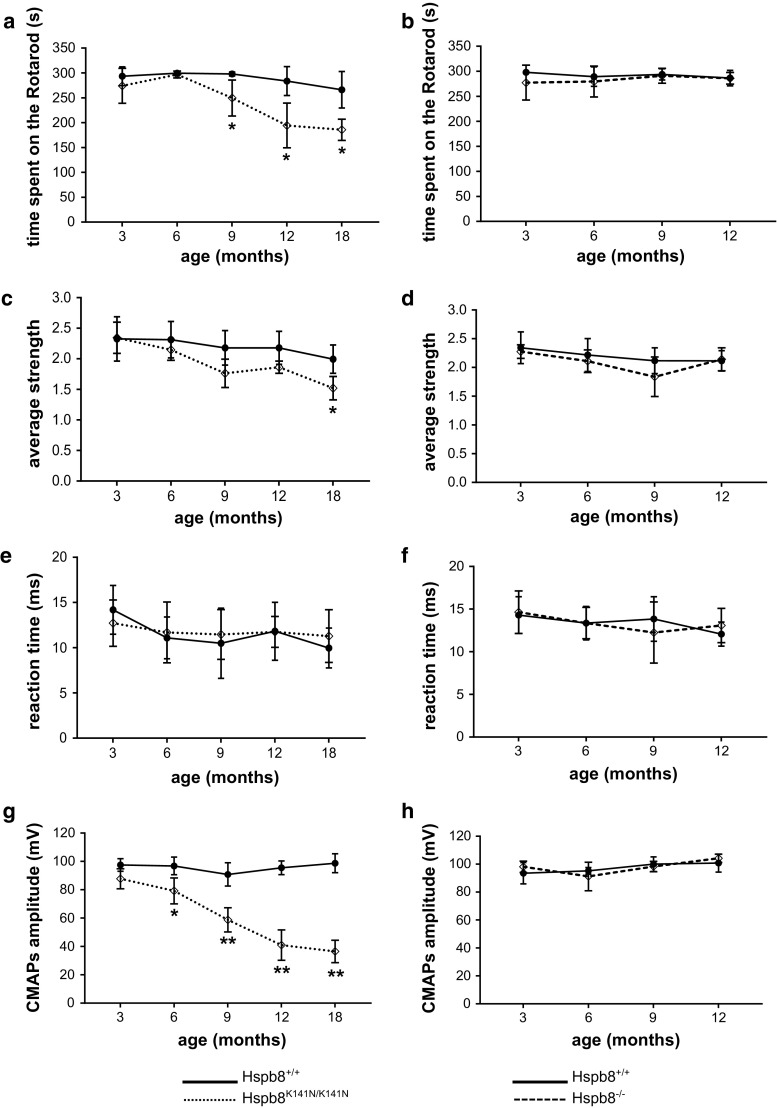
Assessment of motor and sensory functions in the Hspb8^K141N^ KI and Hspb8 KO lines. **a**, **b** Rotarod performance showing the average time, in seconds (s), spent on the accelerating rotating rod at 3, 6, 9, 12, and 18 months of age in Hspb8^K141N/K141N^ mice and their WT littermates (**a**) and at 3, 6, 9, and 12 months of age in Hspb8^−/−^ mice and their WT littermates (**b**). **c**, **d** Average score on the grip strength over time in Hspb8^K141N/K141N^ mice and their WT littermates (**c**) and in Hspb8^−/−^ mice and their WT littermates (**d**). **e**, **f** Hind paws sensitivity to heat shown by the average reaction time, in milliseconds (ms), before showing a sign of discomfort in Hspb8^K141N/K141N^ mice and their WT littermates (**e**) and in Hspb8^−/−^ mice and their WT littermates (**f**). **g**, **h** Average CMAPs amplitude in Hspb8^K141N/K141N^ mice and their WT littermates (**g**) and in Hspb8^−/−^ mice and their WT littermates (**h**). Values are expressed as mean ± SD, *N* = 9. *p<0.05, and **p<0.01

The Hspb8^K141N/K141N^, Hspb8^K141N/+^, Hspb8^−/−^, and Hspb8^−/+^ mice and their WT littermates were screened for motor or sensory deficits at 3, 6, 9, and 12 months of age. The KI lines (Hspb8^K141N/K141N^, Hspb8^K141N/+^) were also tested at 18 month. None of the mice showed clasping behaviour and no difference in footprint could be detected between the transgenic heterozygous and homozygous KI and KO mice and their respective WT littermates. From the age of 9 months, the homozygous mutant Hspb8^K141N/K141N^ mice showed a progressive and significant decline in the locomotor performance assessed by the Rotarod (Fig. 2a). A decline was also observed at the grip strength test where the Hspb8^K141N/K141N^ mice achieved a significantly lower performance than their WT littermates at 18 month (Fig. 2c). However, the Hspb8^K141N/K141N^ mice score at the hot plate test was normal (Fig. 2e), suggesting that the sensory functions were preserved. The motor deficit was further supported by a drastic and significant reduction of the amplitude of the compound motor action potentials (CMAPs) in the Hspb8^K141N/K141N^ mice from the age of 6 months onwards (Fig. 2g), while the nerve conduction velocities were normal, hinting for axonal loss rather than demyelination of the nerve. On the other hand, the heterozygous Hspb8^K141N/+^ mice performances did not differ from the WT (Supplementary Fig. S1). These mice were, therefore, not included in further analyses. These data demonstrate that homozygous expression of Hspb8^K141N^ mutation induces motor behavioural and electrophysiological impairments without sensory abnormalities similar to the dHMN type II phenotype [75]. Interestingly, neither the homozygous Hspb8^−/−^ mice (Fig. 2) nor the heterozygous Hspb8^−/+^ (Supplementary Fig. S1) developed motor or sensory deficits or CMAPs abnormalities suggesting that part of the motor phenotype observed in the Hspb8^K141N/K141N^ mice is the consequence of a toxic gain-of-function of the mutant protein.

### Hspb8^K141N/K141N^ mice show severe axonal degeneration

**Fig. 3:**
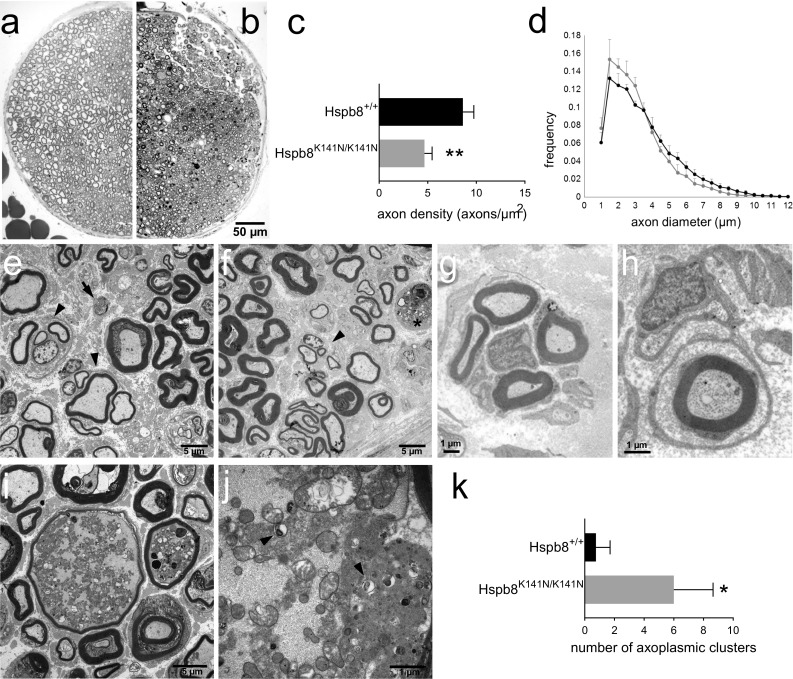
Characterization of distal sciatic nerve pathology associated with Hspb8^K141N^ mutation. **a**, **b** Toluidine-blue stained semi-thin sections of the tibial nerve of 18-month-old WT Hspb8^+/+^ (**a**) and Hspb8^K141N/K141N^ (**b**) mice. **c** Density of large and medium myelinated axons in the tibial nerve of 18-month-old Hspb8^K141N/K141N^ and Hspb8^+/+^ mice. Values are expressed as mean ± SD, *N* = 3. **p<0.01. **d** Axon diameter distribution. Values are expressed as mean ± SD, *N* = 3. **e**, **f** Electron microscopy images of the tibial nerve of 18-month-old Hspb8^K141N/K141N^ mice showing axonal degeneration (*arrow*), regenerative clusters (*arrow head*), and macrophages (*asterisk*). **g**, **h** Electron microscopy images of the sural nerve of the CMT-M:V.6 patient showing a regenerative cluster (**g**) and an onion-bulb like pattern (**h**). **i** Electron microscopy images of the tibial nerve of Hspb8^K141N/K141N^ mice showing axoplasmic accumulation of osmiophilic material. **j** Higher magnification micrograph of the accumulated axoplasmic material showing mitochondria undergoing mitophagy (*arrow heads*). **k** Total number of axoplasmic clusters per nerve cross section in Hspb8^K141N/K141N^ mice and controls. Values are expressed as mean ± SD, *N* = 3. *p<0.05

To determine if the reduced CMAPs amplitude was associated with axonal degeneration, we quantified the number of myelinated axons on Toluidine blue-stained semi-thin cross sections of the distal sciatic nerve (tibial nerve) of 18-month-old Hspb8^K141N/K141N^ mice (Fig. 3b) and their WT littermates (Fig. 3a). The densities of large and medium myelinated axons were significantly lower in the Hspb8^K141N/K141N^ mice compared to their WT littermates (Fig. 3c, d), similar as seen in the severe peripheral axon loss reported in the postmortem neuropathological analysis of a dHMN patient [58]. Quantification of the large neurons in the ventral horn of the spinal cord showed that despite the severe distal axon loss, the motoneuron cell bodies were spared (Supplementary Fig. S2a, b). Importantly, the reduction in axon density seen in 18-month-old Hspb8^K141N/K141N^ mice could not be detected in 2-month-old mice (Supplementary Fig. S2c–e). This led us to attribute the axon loss in the Hspb8^K141N/K141N^ mice to progressive axonal degeneration rather than a developmental defect. In 12-month-old Hspb8^K141N/+^ mice (Supplementary Fig. S3a–c) and Hspb8^−/−^ mice (Supplementary Fig. S2f–h), similar analyses failed to demonstrate axon loss, supporting the absence of neuropathy in these mice. At the ultrastructural level, distal sciatic nerves of 18-month-old Hspb8^K141N/K141N^ mice demonstrated histopathological changes such as axonal atrophy, regenerative clusters, and infiltration by macrophages (Fig. 3e–f). In addition, Hspb8^K141N/K141N^ mice sciatic axons displayed focal accumulations of mitochondria, some of them undergoing mitophagy, as well as other degenerating organelles (Fig. 3i, j). This accumulation of axoplasmic material has already been described in a case of CMT2E neuropathy caused by *NEFL* gene mutations [26, 84] and is thought to result from axoplasmic flow disturbance. These clusters of axoplasmic degenerative material were significantly more frequent in Hspb8^K141N/K141N^ than in WT mice (Fig. 3k). Interestingly, the distal sciatic nerve of heterozygous Hspb8^K141N/+^ mice also displayed, although to a lesser extent, regenerative clusters, macrophage infiltration, and accumulation of axoplasmic material (Supplementary Fig. S3d, e), as well as myelinated Remak bundles which suggest abnormal cycles of regeneration and remyelination (Supplementary Fig. S3f). It is so far difficult to determine if the sciatic nerve of HSPB8-linked dHMN II patients shows a similar pattern because of the absence of motor nerve biopsies. However, a biopsy of the sensory branch of the superficial peroneal nerve of one patient belonging to the large Belgian family with the HSPB8_K141N mutation (patient CMT-M:V.6) was analysed and no major abnormalities were found in the ultrastructure apart from a regenerative cluster (Fig. 3g), three small Schwann cell onion bulb-like formations (Fig. 3h), and a minimal reduction in unmyelinated axons [77].

### Hspb8^K141N/K141N^ mice develop muscle atrophy with myofibrillar alterations

**Fig. 4:**
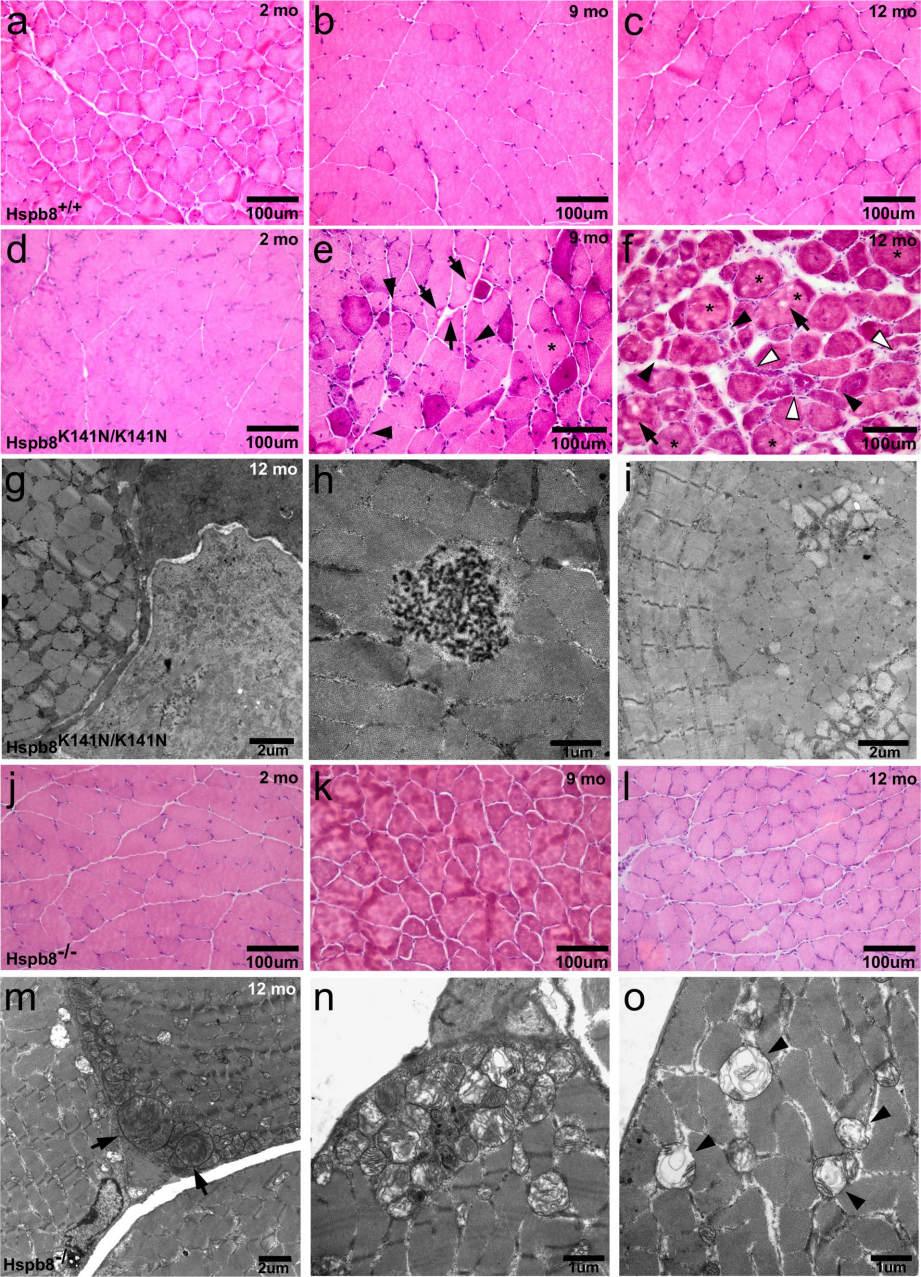
Gastrocnemius muscle histopathology in the Hspb8^K141N^ KI and Hspb8 KO lines. **a**, **c** Haematoxylin and eosin (H&E)-stained cross sections of the gastrocnemius muscle of 2-month-old (**a**), 9-month-old (**b**) and 12-month-old (**c**) Hspb8^+/+^ mice. **d**, **f** H&E-stained cross sections of the gastrocnemius muscle of 2 month old (**d**), 9-month-old (**e**), and 12-month-old (**f**) Hspb8^K141N/K141N^ mice. **e**, **f** Rimmed vacuoles (*black arrows*), necrotic (*white arrowheads*), atrophied (*black arrowheads*), and hypertrophic fibers (*asterisks*) in 9-month-old (**e**) and 12-month-old (**f**) Hspb8^K141N/K141N^ mice. **g**, **i** Electron microscopy images of the gastrocnemius muscle of a 12-month-old Hspb8^K141N/K141N^ mouse showing myofibers at different stages of atrophy (**g**); accumulation of granulofilamentous material (**h**) and a ring fiber showing classical features with peripheral longitudinal myofibrils and centrally transverse myofibrils (**i**). **j**, **l** H&E-stained cross sections of the gastrocnemius muscle of 2-month-old (**j**), 9-month-old (**k**) and 12-month-old (**l**) Hspb8^−/−^ mice. **m–o** Electron microscopy images of the gastrocnemius muscle of a 12-month-old Hspb8^−/−^ mouse showing intact myofibrils and normal Z-disc organisation (**m**), enlarged mitochondria with altered cristae (*arrow*) (**m**), accumulation of altered mitochondria, some possibly undergoing mitophagy (**n**), and mitochondria presenting degenerating cristae and abnormal matrix (*arrow heads*) (**o**)

The peroneal brevis muscle biopsy of the same CMT-M:V.6 patient showed a total fatty degeneration with only few residual atrophic muscle fibers [77]. This led us to proceed with the histopathology of a hind limb distal muscle innervated by the sciatic nerve, the gastrocnemius muscle. Haematoxylin and eosin (HE) staining of transversal sections of the gastrocnemius muscle showed that the myofibers of 2-month-old Hspb8^K141N/K141N^ (Fig. 4d) and WT mice (Fig. 4a) were of regular size and displayed a normal morphology with most nuclei positioned at the normal subsarcolemmal location. In contrast, the 9-month-old Hspb8^K141N/K141N^ mice (Fig. 4e), but not their WT littermates (Fig. 4b), displayed an increased number of myofibers with non-subsarcolemmal nuclei, a marked discrepancy in myofiber size, some of them being atrophic, and several rimmed vacuoles. In the 12-month-old Hspb8^K141N/K141N^ mice gastrocnemius muscle, internalized nuclei were more frequent, size variability was markedly increased, and angulated (but non-grouped), and rounded atrophic fibers could be seen as well as hypertrophic fibers, clumped nuclei, rimmed vacuoles, and endomysial fibrosis (Fig. 4f). Notably, the myofibers of 12-month-old WT mice also showed some size variability (Fig. 4c), but much less pronounced than in Hspb8^K141N/K141N^ mice, reflecting the normal aging process of skeletal muscle in these mice. Interestingly, the fiber-type pattern of the Hspb8^K141N/K141N^ mice was not grouped and was similar to WT littermates (Supplementary Fig. S4e–f), suggesting that the muscle atrophy had a myogenic rather than neurogenic origin. The myopathic phenotype in Hspb8^K141N/K141N^ mice was further validated and refined by EM analyses showing severe myofiber degeneration (Fig. 4g), accumulations of electron dense granulofilamentous material (Fig. 4h), and ring fibers (Fig. 4i); these features are often associated with myofibrillar myopathy (MFM) [17]. These data echo the recent description of a MFM-like phenotype in two families with the HSPB8 (c.421A > G;p.K141E) mutation [34]. The H&E-stained muscle sections of 12-month-old heterozygous Hspb8^K141N/+^ mice were comparable to WT (Supplementary Fig. S3 g, h), while EM analysis revealed signs of Z-band disintegration (Supplementary Fig. S3i), accumulation of electron-dense material (Supplementary Fig. S3j), and a small, empty vacuole (Supplementary Fig. S3 k). Similar histopathological studies were carried out in the KO lines and showed a subtle increase in myofiber size variability in Hspb8^−/−^ mice (Fig. 4j–l) with age comparable to what was seen in WT animals. Further EM analyses in the gastrocnemius of Hspb8^−/−^ mice showed the absence of atrophic or necrotic fibers, preserved Z-disc structure (Fig. 4m), and accumulation of pathologic mitochondria many of which presented with degenerating cristae and abnormal matrix (Fig. 4n, o). These data suggest that loss of function of Hspb8 may affect mitochondrial homeostasis, but does not lead to myofibrillar myopathy, while mutant Hspb8 causes progressive and severe myopathy with a distinct myofibrillar component.

### Mutant Hspb8 causes aggregation of myofibril components and autophagy defects in the muscle

**Fig. 5:**
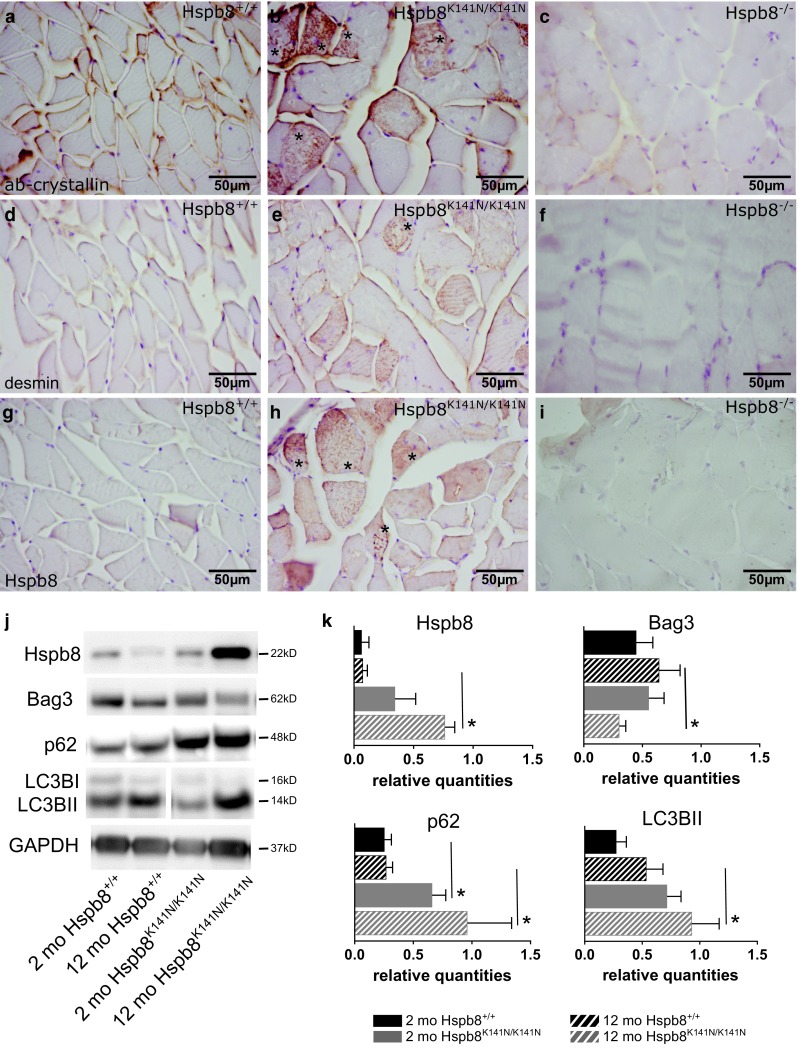
Aggregates of myofibrillar components and autophagy defects in the muscle of Hspb8^K141N/K141N^ mice. **a**–**c** αB-crystallin immunohistochemistry in 12-month-old Hspb8^+/+^ (**a**), Hspb8^K141N/K141N^ (**b**) and Hspb8^−/−^ (**c**) mice, with notable dense subsarcolemmal and intermyofibrillar aggregates in Hspb8^K141N/K141N^ mice (**b**). **d**, **f** Desmin immunohistochemistry in 12-month-old Hspb8^+/+^ (**d**), Hspb8^K141N/K141N^ (**e**) and Hspb8^−/−^ (**f**) mice showing aggregates (asterisk) in some myofibers of Hspb8^K141N/K141N^ mice (**e**). **g**, **h** Hspb8 immunohistochemistry in 12-month-old Hspb8^+/+^ (**g**), in 12-month-old Hspb8^K141N/K141N^, showing numerous myofibers containing aggregates (**h**), and in 12-month-old Hspb8^−/−^ (**i**). **j** Western blot showing Hspb8, Bag3, p62, and LC3B in muscle lysates of 2-and 12-month-old Hspb8^K141N/K141N^ and WT mice. GAPDH is used as a loading control. **k** Quantification of the Hspb8, Bag3, p62, and LC3BII bands normalized on GAPDH. *p < 0.05. Values are expressed as mean ± SD, *N* = 3

Because aggregation of myofibrillar or ectopic proteins is a major hallmark of MFM [47], we looked for desmin and αB-crystallin, also known as HSPB5 or CRYAB, in the muscle of 12-month-old Hspb8^K141N/K141N^ mice and their WT littermates. While αB-crystallin immunoreactivity was found sub-sarcolemmaly and colocalizing with the Z-disc in the WT animals (Fig. 5a), large aggregates were observed in many muscle fibers of the Hspb8^K141N/K141N^ mice (Fig. 5b). Similar patterns were observed for desmin (Fig. 5d, e). No αB-crystallin and desmin aggregates were found in the 12-month-old Hspb8^−/−^ mice (Fig. 5c, f) which corroborates with the absence of an MFM phenotype in these mice. As expected, Hspb8 forms also aggregates in Hspb8^K141N/K141N^ mice (Fig. 5h), while it follows a certain sarcomeric structure in WT (Fig. 5g). Comparable aggregates could not be found in Hspb8^K141N/+^ mice (Supplementary Fig. S3l). As expected, no Hspb8 immunoreactivity could be seen in KO tissue (Fig. 5i). Taken together, these findings are in concordance with the HSPB8, desmin, and αB-crystallin aggregates found in the patients with a different mutation in the same lysine residue, the K141E missense mutation [34]. To expand our understanding of the disease mechanism, we proceeded with protein expression analyses of Hspb8 and its main interactor Bag3 in 2-month-old (pre-symptomatic) and 12-month-old (symptomatic) mice. In addition, we also quantified the protein levels of p62, a substrate for autophagy, and of the autophagosome-associated lipidated form of LC3B (LC3BII), both used as common markers for autophagy. Compared to WT littermates, the muscle of symptomatic Hspb8^K141N/K141N^ mice displayed a significant increase in HSPB8, p62, and LC3BII, while Bag3 levels were decreased (Fig. 5j, k). For p62, an increase could also be observed in 2-month-old Hspb8^K141N/K141N^ mice. The increase in Hspb8 level observed in 12-month-old Hspb8^K141N/K141N^ mice correlates with the accumulation of Hspb8 aggregates previously described. The downregulation of the Bag3 protein level in the same mice could suggest a deficit in Bag3-mediated autophagy. In addition, the significant increase in p62, a substrate of autophagy, in 2-month-old and 12-month-old Hspb8^K141N/K141N^ mice (Fig. 5j, k) could reflect a failure in autophagy. The increase in LC3BII, a marker of autophagosomes, could either reflect an increase in autophagy flux, which is somewhat incompatible with the higher p62 and lower Bag3 levels, or reflect an accumulation of autophagosomes that fail to fuse with lysosomes, a known consequence of the missense mutation in HSPB8 [50].

### Mutant Hspb8 forms aggregates and causes low autophagy power in the sciatic nerve

**Fig. 6:**
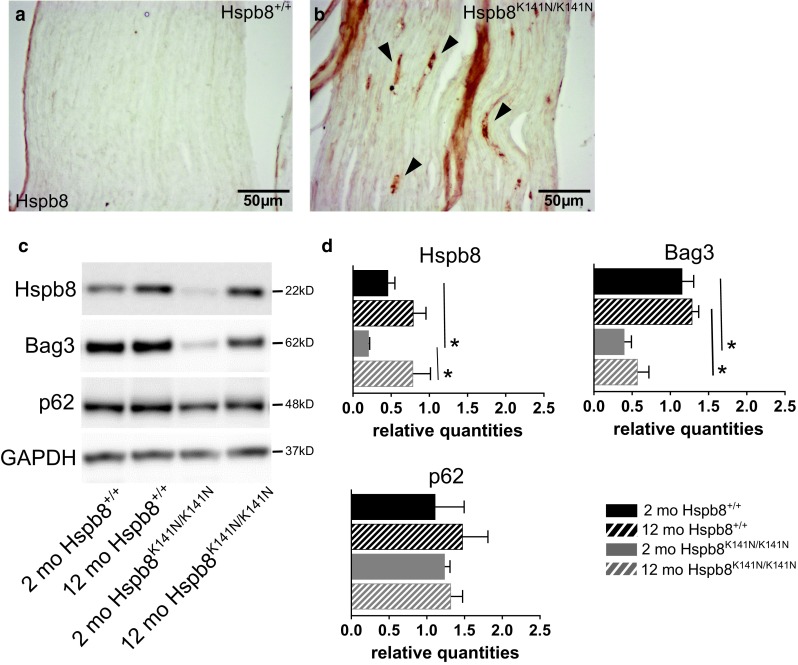
Hspb8 aggregates and low autophagy power in the sciatic nerve of Hspb8^K141N/K141N^ mice. **a**, **b** Hspb8 immunohistochemistry in 18-month-old WT (**a**) and Hspb8^K141N/K141N^ (**b**) mice showing that only the Hspb8^K141N/K141N^ mice develop aggregates (**b**). **c** Western blot showing Hspb8, Bag3 and p62 and in sciatic nerve lysates of 2-and 12-month-old Hspb8^K141N/K141N^ and WT mice. GAPDH is used as a loading control. **d** Quantification of the Hspb8, Bag3, and p62 bands normalized on GAPDH. *p < 0.05. Values are expressed as mean ± SD, *N* = 3

Similar to the muscle, we found Hspb8-positive aggregates in symptomatic 18-month-old Hspb8^K141N/K141N^ mice (Fig. 6b) but not in Hspb8^K141N/+^ mice (supplementary Fig. S3m) or WT littermates (Fig. 6a). We then measured the amount of proteins involved in Hspb8/Bag3-mediated autophagy in 2-month-old and 12-month-old mice by western blot. The levels of Hspb8 and Bag3 were significantly reduced in the sciatic nerve of 2-month-old Hspb8^K141N/K141N^ mice (Fig. 6c, d). The level of Bag3 was also drastically reduced in 12-month-old Hspb8^K141N/K141N^ mice, whereas Hspb8 protein level was comparable to WT. The levels of p62 were similar to WT both in pre-symptomatic and symptomatic Hspb8^K141N/K141N^ mice. The lipidated form of LC3B could not be detected in sciatic nerve tissue. The absence of detectable LC3BII band was specific to the sciatic nerve, since we could detect it in other tissues from the same animals (Supplementary Fig. S5). We cannot explain this variability in LC3BII detection. One hypothesis could be that the level of autophagy or/and the speed of degradation of autophagosomes (where LC3BII is located) varies among tissues. The affected Hspb8 and Bag3 protein levels in the absence of p62 and LC3 alteration is reminiscent of the low “autophagy power” pattern described by Crippa et al. in the spinal cord of mutant SOD1 mice [18]. This reduced potential to activate the Hspb8/Bag3-mediated macroautophagy pathway could contribute to the pathogenesis by impairing the degradation of the mutant Hspb8 protein that accumulates and progressively forms insoluble toxic aggregates.

## Discussion

We generated a new transgenic mouse model for the Hspb8^K141N^ mutation causative of dHMN type II, CMT2L, and distal myopathy. The previous transgenic mouse models of mutant Hspb8 have been published [23, 66, 88], but this one is the first with a physiological and ubiquitous expression of the mutant protein. It is the first report of an Hspb8 transgenic mouse line modelling distal myopathy and the faithful reproduction of a human hereditary neuromuscular disease phenotype by a mouse model is actually a rather rare event [6].

It is not entirely clear how independent the neuropathic and myopathic phenotypes are in our Hspb8^K141N/K141N^ mice. The histopathological analysis of the gastrocnemius muscle showing normal fiber type pattern, granulofilamentous material accumulation, Z-disc disorganisation, and cytoskeletal component aggregates points toward a myopathy with myofibrillar component, distinct from a neurogenic muscle atrophy. On the other hand, the muscle, the nerve, and the motor neurons are the components of the same neuromuscular axis. They are interdependent and are also particularly dependent on a well-functioning proteostatic system. Denervation leads to major changes in proteostasis in the affected muscle fibers, including myofibrillar remodeling and target formation, and induces a dynamic response involving chaperons, endoplasmic reticulum, and autophagy-related proteins [44]. Mutation in these proteins will affect the motor unit’s ability to respond to denervation and might trigger a disproportionate “bystander” myopathy. This is supported by the observation that mutations in the same proteins can cause both neuropathies, motor neuron diseases and vacuolar myopathies [5, 25, 34, 44, 45, 56, 72, 76].

The heterozygous Hspb8^K141N/+^ mice, which have an allelic pattern closer to the patient situation, have a much milder phenotype and fail to develop motor deficits. The need of a higher expression of the mutant protein to observe a functional phenotype is not peculiar when modelling inherited disease [7, 88] and underlines the discrepancies between transgenic mice and human patients, between their respective lifespan, the disease duration, and the complexity of their environment.

Our model allowed the generation of a functional KO line in parallel with the Hspb8^K141N^ KI lines. An Hspb8 KO mouse model has already been developed by others to determine the function of Hspb8 in cardiac tissues, where it is highly expressed [61]. Although that Hspb8 KO had a normal behaviour and physiology, they showed an increased susceptibility to heart failure under the specific context of cardiac overload [61]. Our Hspb8 KO (Hspb8^−/−^) mice did not develop a neuropathic or myopathic phenotype. The absence of myofiber defect was unexpected, since HSPB8 participates in myofibril stabilisation through the CASA complex [38, 79, 80]. Furthermore, depletion of Hspb1, another member of the sHSP family whose mutant form has also been associated with myopathy, leads to myofiber ultrastructural defects [46]. Although the Hspb8^−/−^ myofibers did not undergo atrophy and degeneration, they presented an accumulation of abnormally patterned mitochondria. Interestingly, comparable structural abnormalities of mitochondria have already been associated with myopathy [57, 83]. This particular susceptibility of mitochondria to Hspb8 deficiency could be attributed to the involvement of Hspb8 in mitochondrial membrane potential maintenance and oxidative phosphorylation [42, 52, 62]. Still, the absence of a phenotype in our KO mice demonstrates that Hspb8 loss-of-function is insufficient to produce a strong neuropathic or myopathic phenotype. This implies that a toxic gain-of-function of mutant Hspb8 plays a key role in the pathogenesis of both diseases in our mouse model. However, a recent report describes several patients with an HSPB8 frameshift mutation leading to haploinsufficiency associated with a late (adult) onset myopathy but not with neuropathy [25]. This suggests that some loss-of-function mechanisms also play a role in the development of HSPB8-associated myopathy.

The toxicity of mutant Hspb8 could result from its tendency to accumulate and form large aggregates in the sciatic nerve and myofibers of Hspb8^K141N/K141N^ mice. We have previously shown that mutant Hspb8^K141N^ is prone to aggregation in patient-derived material [42]. While a few additional studies have associated mutant protein aggregation to axonal neuropathy [67, 78, 87], there is thus far no clear consensus on how these aggregates contribute to the neurodegeneration. Myofibrillar myopathy (MFM) is also caused by aggregation-prone mutations in proteins associated with the Z-disc [4, 10, 53, 71], including αB-crystallin, SQSTM1, and BAG3, which interact with HSPB8 to mediate chaperone-assisted-selective autophagy (CASA), a process required for the maintenance of myofibrils.

The MFM phenotype associated with BAG3 mutation is thought to result from the progressive accumulation of insoluble BAG3 aggregates and the impairment of the CASA complex to remove these aggregates [49, 64]. In parallel, the neuropathic and myopathic phenotypes in the Hspb8^K141N/K141N^ mice could result from the progressive aggregation of mutant Hspb8 and a reduced ability to remove these aggregates. Indeed, the accumulation of aggregate-prone proteins can be prevented by Hspb8 and Bag3 [12, 16, 20, 21], whose levels are affected in the sciatic nerve of our KI mice. In the muscle, the increase in p62 in post-symptomatic mice suggests a defect in the autophagic flux. The higher levels of LC3BII detected in post-symptomatic Hspb8^K141N/K141N^ mice compared to WT could result from the accumulation of autophagosomes unable to fuse with lysosomes and complete the autophagy flux, since mutant Hspb8^K141N^ was shown to impair the fusion of autophagosomes with lysosomes [50].

Altogether, our results lead us to conclude that the accumulation of mutant Hspb8^K141N^ combined with an inability to degrade Hspb8-positive aggregates through Hspb8/Bag3-mediated autophagy is a key pathomechanism underlying the peripheral neuropathy and distal myopathy in our mouse model. Accumulation of toxic protein clusters within the neuronal soma could be tolerated; however, toxic protein accumulation within the confined long peripheral axons could on long-term disturb axoplasmic flow to the distal axon and synapse. Finally, our study demonstrates that the generation of KI and KO mouse lines is a relevant and powerful tool to understand the gain- and loss-of-function mechanisms taking part in the pathogenesis of neuromuscular diseases.

## Electronic supplementary material

Below is the link to the electronic supplementary material.

Supplementary Figure S1 Assessment of motor and sensory functions in the heterozygous Hspb8 KI and KO mice. **a-b** Rotarod performance showing the average time spent on the accelerating rotating rod at 3, 6, 9, 12, and 18 months of age in Hspb8^K141N/+^, Hspb8^K141N/K141N^ and WT littermates (**a**) and at 3, 6, 9, and 12 months of age in Hspb8^-/+^, Hspb8^-/-^, and WT littermates (**b**). **c-d** Average score on the grip strength over time in Hspb8^K141N/+^, Hspb8^K141N/K141N^, and WT littermates (**c**) and in Hspb8^-/+^, Hspb8^-/-^, and WT littermates (**d**). **e–f** Hind paws sensitivity to heat shown by the average reaction time before showing a sign of discomfort in Hspb8^K141N/+^, Hspb8^K141N/K141N^, and WT littermates (**e**) and in Hspb8^-/+^, Hspb8^-/-^, and WT littermates (**f**). **g–h** Average CMAPs amplitude in Hspb8^K141N/+^, Hspb8^K141N/K141N^, and WT littermates (**g**) and in Hspb8^-/+^, Hspb8^-/-^, and WT littermates (**h**). Values are expressed as mean ± s.d.. *N*=9 (EPS 254 kb)

Supplementary Figure S2 **a** Cresyl violet-stained large neurons in the ventral horn of a 58-week-old Hspb8^K141N/K141N^ mouse spinal cord. **b** Number of large neurons in the ventral horn of 58-week-old Hspb8^K141N/K141N^ and Hspb8^+/+^ mice. Values are expressed as mean ± s.d.. *N*=3. **c–d** Toluidine-blue stained semi-thin sections of the tibial nerve of 8-week-old WT (**c**) and Hspb8^K141N/K141N^ mice (**d**). **e** Density of large and medium myelinated axons in the tibial nerve of 8-week-old Hspb8^K141N/K141N^ and WT mice. Values are expressed as mean ± s.d.. *N*=3. **f** Axon frequency per diameter. Values are expressed as mean ± s.d.. *N*=3. **g-h** Toluidine-blue stained semi-thin sections of the tibial nerve of 52-week-old WT Hspb8^+/+^ (**g**) and Hspb8^-/-^ mice (**h**). **i** Density of large and medium myelinated axons in the tibial nerve of 52-week-old Hspb8^-/-^ and WT mice. Values are expressed as mean ± s.d.. N=3. **j** Axon frequency per diameter. Values are expressed as mean ± s.d.. *N*=3 (EPS 7528 kb)

Supplementary Figure S3 **a-b** Toluidine-blue stained semi-thin sections of the tibial nerve of 18-month-old WT Hspb8^+/+^ (**a**) and Hspb8^K141N/+^ (**b**) mice. **c** Density of large and medium myelinated axons in the tibial nerve of 18-month-old Hspb8^K141N/+^ and Hspb8^+/+^ mice. Values are expressed as mean ± s.d.. N=3. Electron microscopy images of the tibial nerve of 18-month-old Hspb8^K141N/+^ mice showing regenerative cluster (arrow) (**d**), axoplasmic accumulations of osmiophilic material (asterisk) (**e**) and abnormal Remak bundle ensheathed by a thin myelin layer (**f**). **g–h** H&E-stained cross sections of the gastrocnemius muscle of 2-month-old WT (**g**) and Hspb8^K141N/+^ mice (**h**). **i–k** Electron microscopy images of the gastrocnemius muscle of a 12-month-old Hspb8^K141N/+^ mouse showing Z-band disintegration (**i**); accumulation of electron-dense material (arrow) (**j**) and an empty vacuole (asterisk) (**k**). Hspb8 immunohistochemistry of the muscle (**l**) and the sciatic nerve (**m**) of 12-month-old Hspb8^K141N/+^ (EPS 27760 kb)

Supplementary Figure S4 **a–f** Cross sections of the gastrocnemius muscle of 12-month-old Hspb8^+/+^ mice (**a–c**) and Hspb8^K141N/+^ mice (**d–f**) stained with ATPase at pH 4.2 (**a,d**); pH 4.6 (**b,e**); and pH 9.4 (**c,f**), respectively (EPS 64483 kb)

Supplementary Figure S5 **a** Western blot showing the two bands of LC3B, corresponding to its lipidated form (14kD) and its non-lipidated form (16kD), in different nervous (sciatic nerve, spinal cord, and brain) and muscle tissues (gastrocnemius muscle, heart) of WT animals (EPS 99 kb)
